# Differential Associations of Cognitive Emotion Regulation Strategies with Depression, Anxiety, and Insomnia in Adolescence and Early Adulthood

**DOI:** 10.3390/ijerph20105857

**Published:** 2023-05-18

**Authors:** Andrea Zagaria, Mariacarolina Vacca, Silvia Cerolini, Michela Terrasi, Valeria Bacaro, Andrea Ballesio, Chiara Baglioni, Philip Spinhoven, Caterina Lombardo

**Affiliations:** 1 Department of Psychology, Sapienza University of Rome, 00185 Rome, Italy; 2 Department of Psychology, University of Bologna, 40136 Bologna, Italy; 3 Department of Human Sciences, University of Rome Guglielmo Marconi, 00193 Rome, Italy; 4 Department of Psychiatry and Psychotherapy, Medical Center, University of Freiburg, 79098 Freiburg, Germany; 5 Institute of Psychology, Leiden University, 2333 AK Leiden, The Netherlands

**Keywords:** cognitive emotion regulation strategies, emotion regulation, insomnia, depression, anxiety, internalizing symptoms

## Abstract

While difficulties with emotion regulation (ER) are consistently linked to poor mental health in adulthood, findings in adolescence have been more mixed. Cognitive ER strategies, which involve the ability to manage emotions through mental processes, may be particularly important during different stages of development due to age-specific adjustments. We conducted two exploratory and cross-sectional studies to examine the relationships between cognitive ER strategies and mental health (i.e., depressive, anxiety, and insomnia symptoms) in two samples: 431 young adults (M_age_ = 20.66 ± 2.21; 70% women and 30% men) and 271 adolescents (M_age_ = 14.80 ± 0.0.59; 44.6% girls and 55.4% boys). The participants completed a group of questionnaires, including the Cognitive Emotion Regulation Questionnaire, the Insomnia Severity Index, the Beck Depression Inventory-II, the State–Trait Anxiety Inventory, and the Youth Self Report. We employed hierarchical multiple regressions to assess the unique contribution of cognitive ER strategies to mental health outcomes. Maladaptive strategies (such as rumination and catastrophizing) were consistently associated with impaired mental health in both samples, while adaptive strategies (such as positive refocusing and positive reappraisal) were only associated with better mental health in young adults. These findings support the importance of cognitive ER strategies as potential risk factors for psychopathology and suggest that interventions aimed at improving emotion regulation may be beneficial. The age-specific differences in the relationship between cognitive ER strategies and mental health may reflect the refinement of emotion regulation abilities across the lifespan.

## 1. Introduction

During the lifetime, the individual ability to regulate emotions is fundamental for mental health and social functioning [1,2,3]. This ability consists of monitoring and managing emotional experiences and responses, using a set of cognitive and behavioral strategies [4]. These emotion regulation (ER) strategies may be defined as adaptive or maladaptive: if used systematically, they can facilitate or hinder the functioning of individuals, protecting them from risk or increasing the risk of adverse outcomes. In adulthood, the relationship between the use of maladaptive emotion regulation (ER) strategies and anxiety [5,6,7] and depression [8,9,10,11] is well documented.

A meta-analytic study by Aldao and colleagues [5], including 114 studies, examined the relationship between ER and psychopathology. The results showed that the use of maladaptive ER strategies such as rumination, suppression, and avoidance was mostly associated with psychopathological outcomes (i.e., anxiety, depression, and eating disorders). Inversely, the use of adaptive ER strategies such as cognitive re-evaluation and problem-solving was associated with a lower prevalence of anxiety, depression, and eating disorders. Another meta-analytic study [12] shows that higher use of the ER strategy of cognitive reappraisal was associated with indicators of higher mental health (i.e., life satisfaction and positive affect) and with indicators of lower mental distress (i.e., depression, anxiety, and negative affect). The opposite pattern of correlations was found for expressive suppression: it was associated with lower mental health and well-being. Other evidence comes from studies confirming that the use of rumination contributed to the genesis and maintenance of depressive disorder in adults [13,14], as well as anxious and depressive symptomatology in adolescent samples [15,16]. McLaughlin and Nolen-Hoeksema [17] demonstrated that rumination might be considered a transdiagnostic factor that accounts for the co-occurrence of symptoms of depression and anxiety, highlighting concurrent and prospective associations in two large samples of adults and adolescents. Consistent with these results, a meta-analytic evaluation of the association between ER strategies and anxiety and depression in adolescence (68 effect sizes from 35 studies included) [18] showed that the habitual use of adaptive ER strategies (cognitive reappraisal, problem-solving, and acceptance) was associated with low depressive and anxiety symptoms, whereas the maladaptive ER strategies (avoidance, suppression, and rumination) were related to more depressive and anxiety symptoms.

Difficulties in emotion regulation and the use of maladaptive ER strategies have also been linked to sleep problems and insomnia disorder [19]. Insomnia is commonly defined as a predominant complaint of dissatisfaction with either the quality or quantity of sleep, which is associated with difficulty initiating sleep, difficulty maintaining sleep, and/or early-morning awakening [20]. This disorder is often associated with dysfunctional ER and mental distress (e.g., depression) [21]. Different studies highlighted that adults who have chronic insomnia or report poor sleep quantity/quality use more frequently dysfunctional ER strategies such as rumination, repetitive negative thinking [22], thought control, suppression, and worry [23,24]. The results from an experimental study by Mauss and colleagues [25] showed that poor sleep quality over the past week was linked with decreased cognitive reappraisal ability in a laboratory paradigm. Recently, a study by Cheng and co-workers [26], including a sample of middle-aged and elderly people, found that insomnia was predicted by the high use of maladaptive strategies, such as catastrophizing, rumination, and self-blame, and by low use of refocusing on planning and positive reappraisal.

Heterogenous results have been found in adolescents [27], indicating that youths aged 13–18 years with greater sleep problems reported less problem-solving and greater avoidance, suppression, rumination, and acceptance. However, studies are still scarce to establish firm conclusions. The role of a broader range of ER strategies on sleep difficulties—as well as on other psychopathological symptoms—should be considered to examine the unique role of various and specific ER strategies in predicting these psychological problems, particularly in adolescence and young adulthood. In this perspective, a comprehensive and detailed set of cognitive emotion regulation (ER) strategies were proposed by Garnefski and colleagues [28]. They validated the Cognitive Emotion Regulation Questionnaire (CERQ), which includes nine subscales representing different cognitive ER strategies [28]. The construct of cognitive ER may be defined as the cognitive way of managing emotional information [29] and refers to the mental part of the emotion regulation process [28]. Numerous studies showed significant associations between CERQ dimensions and psychopathological outcomes, such as anxiety and depression, in samples of adults and adolescents [30,31,32,33]. As cognitive ER strategies involve the ability to manage feelings and emotions, one can argue that some differences may exist in regulating emotions through thoughts and cognitions when divergent lifespans are examined [34]. As an illustration, it has been generally assumed that during adolescence, the more advanced cognitive and emotional abilities are being mastered due to age-specific adjustments (e.g., neurological changes) [33,35]. Adolescents may not have access to the same range of ER strategies as adults, either because of insufficient practice in using them or due to immature executive functions and social cognition skills [36]. The development of the executive and social processes involved in ER (e.g., working memory and decision-making) [37] relies on neural structural and functional changes that interact with the urge to negotiate challenging social contexts [38]. Therefore, adolescence is a crucial phase for developing ER strategies, with long-term consequences for future mental health [36]. In this perspective, as the literature suggests that adult psychopathology is nearly always preceded by adolescent disturbances [39], examining the cognitive emotion regulation–mental distress link among youngsters could provide insight into vulnerability factors for psychopathology in general. Effectively, some studies [33] investigated these age-related differences in the use of cognitive ER strategies and their relationships to symptoms of depression, confirming the presence of specific age-related differences. However, these results are preliminary and very little is known about age differences in the use of cognitive ER strategies and insomnia symptoms, necessitating further investigations.

Furthermore, the role of gender in explaining the relationship between ER strategies and psychopathology should also be considered. As highlighted by Susan Nolen-Hoeksema [40], who reviewed and summarized primary studies regarding the relationship among gender, emotion regulation, and psychopathology, women report using almost all types of emotion regulation strategies more frequently compared to men, and this is consistent with studies including children and adolescents. For example, women reported using rumination, reappraisal, acceptance [41], distraction, and avoidance [42] significantly more than men. Moreover, ER strategies seem to be similarly related to psychopathology in women and men, although a difference was observed in the extent to which adaptive strategies have compensatory effects among women with higher levels of maladaptive strategies, but not among men [40]. In addition, women report more rumination than men, partially accounting for greater depression and anxiety in women compared to men, whereas a greater tendency to use alcohol to cope partially accounts for more alcohol misuse in men compared to women [5]. This panoramic view on the role of gender suggests the importance of controlling for established gender differences when examining the relationships between ER strategies and psychopathological outcomes.

In view of the above-discussed information, we conducted two exploratory and cross-sectional studies aimed at investigating the relationships between cognitive ER strategies and symptoms of depression, anxiety, and insomnia in two separate samples of young adults and adolescents. Our investigations accounted for established gender differences in these relationships. Specifically, in Study 1, we sought to examine the unique contribution of each cognitive ER strategy, beyond gender and other ER strategies measured with the CERQ, to anxiety, depression, and insomnia symptoms in a sample of young adults. In Study 2, we aimed to replicate these findings in a younger age group of adolescents. Consistent with prior research, our hypotheses for both studies included the following: (a) positive associations between maladaptive ER strategies (i.e., rumination, catastrophizing, self-blame, and other-blame) and anxiety, depression, and insomnia symptoms; and (b) negative associations between adaptive ER strategies (i.e., positive reappraisal, positive refocusing, acceptance, refocusing on planning, and putting into perspective) and the same psychopathological symptoms.

***STUDY 1***

## 2. Material and Methods

### 2.1. Procedure

Students were invited during university courses at the Department of Psychology to collaborate on a research study about emotion regulation and psychological health. No compensation for participation in the study was offered. At first, the aims and the method of the study were explained to the students and, if they were interested, they signed a detailed informed consent of the study. After providing written informed consent, a battery of self-report questionnaires was administered in large group sessions during their lecture time. Anonymity was guaranteed by not collecting data that could lead back to someone’s identity through the creation of an alphanumeric code. The alphanumeric code also facilitated the anonymous feedback of the results to the participants. This study was conducted in accordance with the ethical standards established in the Helsinki Declaration [43] and was approved by the Institutional Review Board of the Department of Psychology at Sapienza University of Rome.

### 2.2. Participants

A convenience sample composed of 431 young adults aged 18–28 years (M_age_ = 20.66, SD = 2.21) was recruited among the Student Community of Sapienza University of Rome between September and December 2019. Approximately 70% of participants were women, whilst 30% were men. With respect to cohabitation status, 84% of the sample lived with their family, 11.9% of the lived with roommates, 3% lived alone, and 1.1% lived with his/her partner. All participants resided in Italy.

### 2.3. Measures

#### 2.3.1. Sociodemographic Form

Respondents completed a standardized sociodemographic form assessing gender, age, cohabitation status, and country of residence.

#### 2.3.2. Cognitive Emotion Regulation Questionnaire (CERQ)

The Cognitive Emotion Regulation Questionnaire (CERQ) [29] is a 36-item scale designed to assess individual differences in cognitive ER strategies used in response to stressful events. Nine strategies are evaluated: putting into perspective (e.g., I tell myself that there are worse things in life), acceptance (e.g., I think that I have to accept the situation), positive refocusing (e.g., I think of pleasant things that have nothing to do with it), positive reappraisal (e.g., I think I can learn something from the situation), refocus on planning (e.g., I think about how to change the situation), rumination (e.g., I often think about how I feel about what I have experienced), catastrophizing (e.g., I continually think how horrible the situation has been), self-blame (e.g., I feel that I am the one who is responsible for what has happened), and other-blame (e.g., I feel that basically the cause lies with others). Participants are asked to rate how often they have adopted a specific cognitive strategy on a five-point Likert scale, ranging from 1 (almost never) to 5 (almost always). Higher scores indicate greater use of a certain cognitive ER strategy. The Italian version of the CERQ [44] demonstrated strong psychometric properties, maintaining the original nine-factor structure and showing acceptable internal consistencies of its subscales (α ranging from 0.72 to 0.76), as well as significant relationships with convergent measures of emotion regulation. Cronbach’s alpha in our sample was computed for each dimension, ranging from 0.71 to 0.88.

#### 2.3.3. Beck Depression Inventory-II (BDI-II)

The Beck Depression Inventory (BDI-II) [45] is a widely used 21-item inventory designed to assess the presence and the severity of depressive symptoms occurring over the previous 2 weeks (e.g., loss of interest, pessimism, and past failure). Each item is rated on a 4-point Likert scale, ranging from 0 to 3. A total score is calculated by summing all 21 items, where higher scores indicate more severe depressive symptoms. The global assessment score ranges from 0 to 63, where a score of 14 to 19 indicates mild depression, 20 to 28 indicates moderate depression, and 29 to 63 indicates severe depression. The Italian version of the BDI-II [46] highlighted satisfactory internal consistency, test–retest reliability, and significant relationships with convergent measures of depression, along with a good discriminative power between clinical and non-clinical individuals. Cronbach’s alpha in our sample was 0.89.

#### 2.3.4. Insomnia Severity Index (ISI)

The Insomnia Severity Index (ISI) [47] is a brief 7-item tool designed to assess the perceived severity of insomnia symptoms over the past two weeks. Questions assess several facets of sleep, such as problems at the beginning of sleep, interference with daytime functioning, and sleep satisfaction (e.g., How worried/distressed are you about your current sleep problem). A five-point Likert scale is used to rate each item, yielding a total score ranging from 0 to 28. Higher scores indicate more severe sleep difficulties. Adequate psychometric properties have been reported in the Italian population in terms of internal consistency (α = 0.75) and convergent and concurrent validity [48]. Cronbach’s alpha in our sample was 0.83.

#### 2.3.5. State–Trait Anxiety Inventory Trait Version (STAI-T)

The State–Trait Anxiety Inventory [49] is a valid and reliable instrument consisting of two scales measuring two distinct anxiety concepts: state and trait anxiety. Only the trait version, composed of 20 items, was administered in our investigation. Trait anxiety refers to relatively stable individual differences in anxiety proneness apart from temporary fluctuations in state anxiety. Each item is rated on a 4-point Likert scale, ranging from 1 (almost never) to 4 (almost always) (e.g., I worry too much over something that really does not matter). In addition, a total score ranging from 20 to 80 can be obtained by summing all items, where higher scores indicate greater dispositional anxiety. The Italian version of the STAI-T highlighted strong internal consistency (α = 0.90), with almost all items showing good discriminant power, as well as moderate correlations with measures of depression attesting to its concurrent validity [50,51]. Cronbach’s alpha in our sample was 0.91.

### 2.4. Data Analysis

Data were analyzed using IBM SPSS version 23 (IBM Corporation, Armonk, NY, USA).

Primarily, a series of assumption checks were implemented to examine the feasibility of the regression analyses with the current data. First, considering that multivariate outliers could affect the precision of estimation of the regression weights, participants who exhibited a Mahalanobis distance with a x^2^ value significant at *p* < 0.001 were excluded from further analyses [52]. Second, Mardia’s multivariate kurtosis coefficient [53] was calculated to evaluate whether it is reasonable to assume multivariate normality among the continuous variables under study [54]. If the assumption of multivariate normality holds, it can be inferred that each variable follows a normal distribution and that the relationships between pairs of variables, if present, are both linear and homoscedastic [52]. We integrated these findings by visually inspecting histograms of regression-standardized residuals and verifying whether their distribution approximated a normal distribution [55]. Next, we assessed multicollinearity (i.e., perfect multicollinearity occurs when two or more regressors are perfectly linearly related to each other) by calculating the Variance Inflation Factors (VIFs), where a commonly used rule of thumb is that a VIF greater than 10 suggests evidence of serious multicollinearity [55]. Moreover, the Durbin–Watson test [56] was employed to check for autocorrelation in the residuals, where values between 1.5 and 2.2 can be interpreted as indicating the absence of autocorrelation [54]. Lastly, we calculated zero-order correlations between the explanatory variables and the error terms in the models to detect violations of the exogeneity assumption. After satisfying these preliminary assumptions, we conducted three hierarchical multiple regression analyses, one for each outcome under study (i.e., insomnia symptoms; anxiety symptoms; and depressive symptoms). Gender (coded as a dummy variable: 0 = men and 1 = women) was included as a covariate in the first step with forced entry; subsequently, cognitive ER strategies entered in the second step again with forced entry to examine their additional predictive value once the variance explained by gender were partialled out from outcomes. The R-squared was reported as a measure of the proportion of total variance in the dependent variable accounted for by the regression model, and the increment in R-squared was considered as an indicator of the added value of the ER strategies entered in the second step.

## 3. Results

### 3.1. Preliminary Analyses

Firstly, three cases were identified through Mahalanobis distance as multivariate outliers with *p* < 0.001. All outliers were deleted, leaving 428 cases for regression analysis. Subsequently, multivariate normality was assumed as suggested by Mardia’s multivariate kurtosis coefficient less than *p*(*p* + 2) (168) (see [54]). Thus, each continuous variable was supposed to be normally distributed, and the relationships between pairs of them, if present, were considered to be linear and homoscedastic [52]. These findings were in line with the histograms of regression-standardized residuals, which showed a roughly normal curve. Moreover, Variance Inflation Factor (VIF) values were computed, ranging from 1.064 to 1.798. These estimates confirmed the absence of any multicollinearity issue. In addition, the results of the Durbin–Watson test revealed the lack of autocorrelation between residual terms with values between 1.73 and 1.85. Lastly, no significant correlations were found between cognitive ER strategies and the error terms of the regression equations, suggesting no endogeneity issue.

Descriptive statistics of the variables under study are summarized in Table 1. Independent sample t-tests revealed significant differences in self-blame, putting into perspective, anxiety symptoms, and depressive symptoms across gender (see Table 1). Pearson’s product-moment correlation coefficients between predictor and criterion variables are presented in Table 2. Except for acceptance, all cognitive ER strategies showed significant zero-order correlations with mental health outcomes.

### 3.2. Relationships between ER Strategies and Psychopathological Outcomes

Three separate hierarchical regression analyses were conducted to investigate the unique contribution of specific ER strategies on psychopathological outcomes over and above gender differences and each of the other ER strategies. The final steps significantly accounted for approximately 52% of the variance of anxiety symptoms (F(10, 400) = 43.661, *p* < 0.001, R^2^ = 0.522), 34% of the variance of depressive symptoms (F(10, 413) = 21.033, *p* < 0.001, R^2^ = 0.337), and 15% of the variance of insomnia symptoms (F(10, 396) = 7.179, *p* < 0.001, R^2^ = 0.153). Differences between R^2^ and the adjusted R^2^ were small, suggesting that no redundant predictors were inserted in the regression equation. Results are summarized in Table 3.

More specifically, in the hierarchical regression analysis with STAI-T score as the dependent variable, gender was forced into the equation in the first step: R^2^ = 0.029; F(1, 409) = 12.151, *p* < 0.01. The set of cognitive ER strategies was added in the second step: R^2^_change_ = 0.493; F_change_(9, 400) = 45.830, *p* < 0.001. The final step revealed that, once gender was partialled out (B = 2.267; *p* < 0.01), rumination (β = 0.150; *p* < 0.001), catastrophizing (β = 0.252; *p* < 0.001), and self-blame (β = 0.267; *p* < 0.001) were associated with higher levels of trait anxiety. On the contrary, positive refocusing (β = −0.175; *p* < 0.001), positive reappraisal (β = −0.145; *p* < 0.01), and refocus on planning (β = −0.212; *p* < 0.001) were significantly associated with lower scores of trait anxiety.

Similarly, in the hierarchical regression analysis with BDI-II scores as the dependent variable, gender was forced into the equation in the first step: R^2^= 0.021; F(1, 422) = 9.211, *p* < 0.01. Cognitive ER strategies were subsequently added: R^2^_change_ = 0.316; F_change_(9, 413) = 21.891, *p* < 0.001. The final step revealed that, once gender was partialled out (B =1.697; *p* < 0.05), rumination (β = 0.152; *p* < 0.001), catastrophizing (β = 0.195; *p* < 0.001), and self-blame (β = 0.242; *p* < 0.001) were linked to higher levels of depressive symptoms. On the other hand, positive refocusing (β = −0.118; *p* < 0.01) and refocus on planning (β = −0.150; *p* < 0.01) were significantly associated with lower scores of depressive symptoms.

Lastly, a hierarchical regression analysis with ISI score as the dependent variable was carried out. Gender was again included in the first step: R^2^= 0.006; F(1, 405) = 2.538, *p* > 0.05. Cognitive ER strategies were added in the second step: R^2^_change_ = 0.147; F_change_ (9, 396) = 7.653, *p* < 0.001. The final step showed that catastrophizing (β = 0.130; *p* < 0.05) and self-blame (β = 0.181; *p* < 0.001) were significantly related to higher levels of insomnia symptoms. Conversely, putting into perspective (β = −0.109; *p* < 0.05) was significantly associated with lower insomnia symptoms.

## 4. Discussion

The main purpose of this first study was to explore the relationships between cognitive ER strategies and depression, anxiety, and insomnia symptoms in a sample of non-clinical young adults and, more specifically, to evaluate the unique contribution of each specific ER strategy on psychopathological outcomes controlling for gender differences and the other ER strategies. The results from the correlation analyses concurred with previous evidence [5] suggesting that all the maladaptive CERQ strategies correlated with greater psychopathological symptoms, as suggested by the small to moderate associations with depression and anxiety symptoms and the small correlation coefficients observed for sleep symptoms. Moreover, negative small to moderate associations were observed for the adaptive strategies, as expected [57], with the exception of acceptance, which demonstrated no significant correlations with psychopathology, supporting previous works [58,59].

Findings from the regression analyses showed that individual differences in the propensity to experience anxiety and depression symptoms were uniquely linked to rumination, catastrophizing, self-blame, low positive refocusing, and low refocus on planning, supporting the relevance of these processes in cognitive theories of emotional distress [60]. More specifically, positive refocusing and refocusing on planning have consistently resulted to be relevant for beneficial psychological health outcomes (e.g., decreased negative affect) [61]. As regards rumination, metanalytic evidence proposes this strategy to be a transdiagnostic factor associated with both depression and anxiety [62] through a variety of cognitive mechanisms (e.g., perceived uncontrollability of ongoing threats). Results on catastrophizing and self-blame substantiated previous findings in the literature that individuals with expectancies for negative outcomes [63] and attributing undesirable events to their own behavior [64] are more prone to experience emotional distress [5].

The negative unique association found between positive reappraisal and anxiety provides additional support for the beneficial role of these ER strategies in preventing and treating anxious symptoms [65].

However, the result that positive reappraisal did not uniquely predict depression scores did not appear to corroborate previous observations on the link between this cognitive ER dimension and low depressive symptoms [66,67]. For instance, decreased use of positive reappraisal was found to be related to more negative thinking and expectations about the future, which is a known symptom of depression [68]. The apparent lack of a significant role of positive reappraisal in predicting low depression observed in the present study could be related to other underlying mechanisms, such as worries. Namely, previous authors found that low reappraisal was associated with higher depression in individuals high in trait worry but not among low worriers [69], thus encouraging further studies to examine potential moderators and mediators of this relationship. Moreover, it was observed that positive reappraisal was related to increased positive emotion but not decreased negative emotion [70]. One possible explanation for this finding, discussed by previous authors, could be that individuals who identify new positive meanings about the situation and consequently experience positive emotional states may still preserve the negative interpretation of the stressor, and thus demonstrate unchanged levels of negative emotion [70]. Future investigations should include the assessment of positive emotions in addition to depressive symptoms in order to examine the role of reappraisal in differently explaining these outcomes. In conclusion, we should sound a note of caution with regard to the non-significant role of positive reappraisal in depression scores, especially considering that the relative bivariate correlation coefficient (i.e., *r* = −0.280) was notably high as compared to those observed in the literature (e.g., *r* = −0.05) [34].

The results on insomnia showed a unique significant contribution of self-blame and catastrophizing in explaining high ISI scores, substantiating previous findings in the literature [27], and suggesting that people experiencing sleep disturbances are prone to use disengaging coping or emotional-focused coping strategies [71]. One possible mechanism of these associations may involve repetitive negative thinking (RNT) in the form of worry, which reflects the experiencing of future, negative consequences of the current mood state [72]. RNT, considered a transdiagnostic factor associated with the onset and maintenance of a wide range of mental health problems [73], is typically related to sleep problems [74] and may involve dysfunctional emotion regulation or be a manifestation of it [75]. Future studies would investigate the role of worry as a form of RNT in the association between cognitive ER strategies and insomnia.

***STUDY 2***

## 5. Material and Methods

### 5.1. Procedure

The recruitment was performed between October and December 2019. The study was conducted in collaboration between the Department of Psychology of the University of Rome Sapienza and the Department of Human Sciences of the University of Study Guglielmo Marconi of Rome. A total of two high schools in Rome (one in the suburb area of Rome and one in the center of Rome) were selected based on their availability and willingness to respond. At first, the aims and the method of the study were explained to the high-school directors and, if they were interested, they signed a detailed informed consent of the study. After that, a meeting was scheduled with the professors that had the role of class coordinators. In this meeting, the informed consent forms for the parents of adolescents were delivered in order to receive the consent before the administration of the questionnaires to the adolescents. Only adolescents attending the first two years of high school were involved in the study. Once the parent’s consent was obtained, adolescents were asked to sign an informed and written consent and to fill in a battery of questionnaires in their classrooms, with the supervision of their professor. Two different kinds of batteries of questionnaires were created: one for girls and one for boys. All procedures were performed in accordance with the 1964 Helsinki Declaration and its later amendments [43], and the study was approved by the Institutional Review Board of the Department of Psychology at Sapienza University of Rome. Confidentiality and privacy of all participants were guaranteed through the creation of an alphanumeric code affixed to the questionnaires, which could not be paired with the name and surname of participants. These were contained in the informed consent and were kept separate from questionnaires. No compensation for participating in the study was provided.

### 5.2. Participants

This cross-sectional study enrolled 271 adolescents, aged 13–17 years (M_age_ = 14.80, SD = 0.59, 55.4 boys, 44.6% girls). More specifically, 21.2% of the participants had completed their first year of high school, 78.4% had completed their second year of high school, and only 0.4% had completed their third year of high school.

### 5.3. Measures

#### 5.3.1. Sociodemographic Form

Respondents completed a standardized sociodemographic form assessing gender, age, and year of high school attended.

#### 5.3.2. Cognitive Emotion Regulation Questionnaire (CERQ)

See the Measures section of Study 1. Previous studies with adolescent samples have reported satisfactory psychometric properties of the questionnaire [76,77]. In our investigation, except for the catastrophizing dimension that was minimally acceptable (α = 0.69), each subscale showed an acceptable or greater internal consistency with alpha values ranging from 0.72 to 0.88

#### 5.3.3. Insomnia Severity Index (ISI)

See the Measures section of Study 1. In prior research with adolescents, the questionnaire demonstrated satisfactory psychometric properties [78,79]. Cronbach’s alpha in our sample was 0.84.

#### 5.3.4. Youth Self Report (YSR)

The Youth Self Report (YSR) [80] is a widely used questionnaire designed to assess emotional and behavioral problems in youths (age 11–18), covering a wide range of symptom areas. Participants are asked to rate how often they experienced the symptoms or problems during the past six months. Each item is rated on a three-point Likert scale ranging from 0 (not true) to 2 (very true), yielding eight syndrome scores and two higher-order dimensions: internalizing and externalizing. Only the internalizing dimension was considered in the present investigation. The internalizing symptoms score is made up of the withdrawal (e.g., I do not have much energy), somatic complaints (e.g., I feel overtired without good reason), and anxiety/depression (e.g., I cry a lot) syndrome scales. Cronbach’s alpha in our sample was 0.91.

### 5.4. Data Analysis

The same statistical procedures as those used in Study 1 were employed to conduct preliminary analyses and ensure that there were no violations of the key assumptions underlying multiple regression. Two hierarchical multiple regression models were then built to examine if ER strategies had any additional predictive value once the variance explained by gender was accounted for in the outcome variables (i.e., YSR internalizing score and ISI score), and to investigate the unique impact of specific ER strategies on the aforementioned outcomes. Gender was coded as a dummy variable (i.e., 0 = boys; 1 = girls) and was forced into the equation in the first step, while each cognitive ER strategy was added in the second step again with forced entry.

## 6. Results

### 6.1. Preliminary Analyses

Two multivariate outliers among the cases were found using a *p* < 0.001 criterion for Mahalanobis distance. All outliers were deleted, leaving 269 cases for further analysis. Multivariate normality was tenable, as suggested by Mardia’s multivariate kurtosis coefficient less than *p*(*p* + 2) (143) [54]. Hence, continuous variables were assumed to be normally distributed, and relationships between pairs of them, if present, were considered linear and homoscedastic [52]. Consistently, the histograms of regression-standardized residuals showed a roughly normal curve. Moreover, Variance Inflation Factor (VIF) values were between 1.122 and 2.124, suggesting no risk of multicollinearity. In addition, the Durbin–Watson tests, indicating independence of error terms, were between 1.82 and 1.96, and therefore acceptable. Lastly, no significant correlations emerged between cognitive ER strategies and the error terms of the regression equations, suggesting no endogeneity issue.

Descriptive statistics of the variables under study are summarized in Table 4. Independent sample t-tests showed significant differences in rumination, self-blame, other-blame, internalizing symptoms, and insomnia symptoms across gender (see Table 4). Pearson’s correlation coefficients between predictor and criterion variables are summarized in Table 5. Except for other-blame and acceptance, all cognitive ER strategies showed significant zero-order correlations with mental health outcomes.

### 6.2. Relationships between ER Strategies and Psychopathological Outcomes

Two hierarchical multiple regression analyses were performed to assess the unique impact of cognitive ER strategies on internalizing and insomnia symptoms over and above gender differences and the other ER strategies. Determination coefficients from the final steps indicated that close to 46% of the variance of internalizing symptoms (F(10, 211) = 18.187, *p* < 0.001, R^2^ = 0.463) and 29% of the variance of insomnia symptoms F(10, 211) = 8.589, *p* < 0.001, R^2^ = 0.289) were explained by the regression models. Differences between R^2^ and the adjusted R^2^ were relatively small, suggesting that no redundant predictors were inserted in the models. The results are summarized in Table 6.

More specifically, in the hierarchical multiple regression with YSR internalizing score as the dependent variable, gender was forced into the equation in the first step: R^2^ = 0.099; F(1, 220) = 24.298, *p* < 0.001. In the second step, cognitive ER strategies were entered in the model: R^2^_change_ = 0.363; F_change_(9, 211) = 15.866, *p* < 0.001. The final step showed that, once gender was partialled out (B = 5.260, *p*  <  0.001), self-blame (β = 0.259, *p*  <  0.001), rumination (β = 0.214, *p*  <  0.001), and catastrophizing (β = 0.168, *p*  <  0.01) were significantly associated with higher levels of internalizing symptoms.

In the hierarchical multiple regression with ISI score as the dependent variable, gender again was entered in the first step: R^2^ = 0.040; F(1, 220) = 9.225, *p* < 0.01. Cognitive ER strategies were entered in the second step: R^2^_change_ = 0.249; F_change_(9, 211) = 8.216, *p* < 0.001. The final step revealed that, once gender was partialled out (B = 1.253, *p*  <  0.05), rumination (β = 0.236, *p*  <  0.001), and catastrophizing (β = 0.195, *p*  <  0.01) were significantly associated with higher levels of insomnia symptoms.

## 7. Discussion

The current study sought to advance research on cognitive emotion regulation, mental distress, and sleep problems among adolescents. Overall, the results corroborated findings on the relevance of emotion dysregulation in adolescent psychological difficulties [81]. More specifically, positive correlations were found between maladaptive CERQ strategies and internalizing and insomnia symptoms, consistent with previous studies [26,82]. On the other hand, negative relationships were observed with adaptive CERQ strategies, suggesting that the more adolescents engaged in emotion regulation strategies traditionally considered effective, the less they reported indicators of mental distress [5]. The results from the regression analysis add substantially to the ongoing debate on whether maladaptive ER functions should be regarded as a risk factor for psychopathology, a trigger for exacerbation of mental distress symptoms, or the prodromal phase of a mental disorder [61,83]. It was indicated that, among maladaptive CERQ dimensions, rumination and catastrophizing uniquely contributed to both internalizing difficulties and sleep problems and self-blame to internalizing difficulties. These findings align with previous studies on adolescent samples [28,82] and offer compelling evidence for the literature suggesting that such strategies are implicated in the experiencing of negative emotions [3,33]. For example, it was observed that high-catastrophizing adolescents experienced depressive symptoms that were four times higher than low-catastrophizing adolescents [84]. The catastrophizing–internalizing problems link in adolescents was interpreted by some authors as evidence of the overlap between this emotion regulation strategy and mood symptoms, as they were historically described as redundant indicators of negative affect [84]. Further studies should examine the association between catastrophizing and internalizing difficulties controlling for negative affectivity, in order to assess the unique contribution of each factor in explaining symptoms. Indeed, longitudinal research is needed to determine the directions of these mechanisms.

Results on self-blame and rumination could reflect the notion that these specific constructs constitute different facets of a transdiagnostic RNT process [85,86], which has been regarded as a significant predictor of the exacerbation of emotional problems in adolescents [87].

Finally, results showed that, among all the cognitive ER strategies, rumination and catastrophizing were associated with high insomnia scores. This finding lends support to the literature on insomnia phenotypes, which suggests that rumination is a prominent characteristic of adolescents with insomnia symptoms and objective short sleep duration [88]. Moreover, the results are in line with the evidence that adolescents reporting more catastrophizing tended to also report more impaired sleep [89]. A possible explanation could involve the individual propensity to sleep-related concerns when suffering from poor sleep quality [89]. Adolescence is a critical developmental period of life characterized by the emergence of new sleep difficulties (e.g., sleep onset problems) [90]. It was observed that adolescents experiencing sleep disturbances reported catastrophic thoughts when attempting to sleep, most of all regarding concerns about performance and interpersonal aspects of school [91]. It is possible that the new challenges and developmental tasks adolescents encounter contribute to their pathological worry about daily hassles, which exacerbate their sleep patterns. Accordingly, the National Sleep Foundation reported that adolescents typically experience sleep loss, restriction, and deprivation [92], and some authors discussed these problems as consequences of developmental changes in sleep physiology as well as greater sleep need as compared to adults [93].

Contrary to our hypotheses, adaptive ER strategies were not uniquely associated with reduced psychopathological symptoms and insomnia. Previous authors stated that while maladaptive ER strategies generally appear problematic for mental health, adaptive ER strategies show weaker associations with psychological difficulties, possibly because they are more context-dependent than maladaptive ER [6]. For instance, positive reappraisal can only be adaptive when the event can be reformulated [5]. This process assumes that individuals already have the capacity of volitional control [36], which may be not sufficiently developed in adolescence. In this regard, the refinement of ER strategies and their effect on mental health depends on the quantity of emotion-eliciting stressful occasions, which usually grows as the individual grows older [33] and thus may be underdeveloped in adolescence. Consequently, adolescents may employ adaptive strategies more randomly, and this would result in weaker associations with psychopathology [94]. However, the use of adaptive ER between the current samples of adolescents and young adults apparently suggested no particularly remarkable differences. Further investigations are needed to clarify the context-dependency theory of adaptive ER strategies in adolescence and their impacts on psychopathological difficulties, and how these associations differ from those found in other developmental stages.

## 8. General Discussion

Overall, the results of the present work expanded the relevance of emotion regulation strategies as potential risk factors for individual mental distress [81,95]. The hypothesized models were statistically significant, suggesting that cognitive ER strategies were significantly associated with the psychopathological manifestations analyzed. The present investigation expanded previous research exploring the unique contribution of each emotion regulation strategy in predicting mental distress and sleep disturbances in adults and adolescents. From this perspective, the associations of ER strategies with adult anxiety and depression, adolescent internalizing symptoms, and insomnia scores were examined through hierarchical regression models. However, a significant unique contribution was not observed for each of the nine CERQ dimensions, suggesting these aspects showed to be differently relevant for mental health [96]. Indeed, the results on correlation analyses indicated small (*r* ≥ 0.1) to medium associations (*r* ≥ 0.5), suggesting that an important task for future research could be testing alternative hierarchical models taking into account the proportion of variance shared by the CERQ dimensions. Of note, the underlying structure of common ER strategies has been examined in the literature by conducting a meta-analytic examination of relevant ER dimensions, revealing three correlated but distinct factors [97]. Nevertheless, the authors reiterated that existing instruments with subscales assessing the full range of strategies are still of great utility, especially if adaptive strategies are of particular interest, as they were not entirely covered by the three factors identified [97]. Therefore, future studies should assess the utility and validity of multiple inventories of ER strategies by examining the associations of each factor with outcomes of interest.

Taken together, the results of the two studies described above indicated that among all the maladaptive cognitive ER strategies, rumination, catastrophizing, and self-blame were significantly linked to mental distress in young adults as well as in adolescents, consistent with previous works [28,60,61]. However, different from previous results [62,98], blaming others did not uniquely explain variance in psychopathological symptoms. One interpretation of this evidence may point to the attributional-style theory of psychopathology [99], which suggests that maladaptive attributional styles are linked to depression and anxiety [100]. According to this model, psychopathology occurs when negative events are explained with internal attributions. This evidence is supported by studies finding that attributing responsibility for outcomes to one’s own actions (i.e., internal locus of control), which reflects self-blame attitudes, often leads to stress and excessive self-criticism [101]. On the other hand, having an external locus of control, which involves blaming others for one’s misfortune more than oneself, may lead to lesser feelings of mental distress [101]. Moreover, these findings reflected results from Garnefski and Kraaji [95], who found that CERQ other-blame was more weakly associated with psychopathology as compared to other subscales. Some authors argued that the relationship between other-blame and mental adjustment is complex [102]. We speculate that the tendency to misattribute blame for one’s actions to external forces may result in more efficient coping with uncontrollable events in some cases, as individuals easily accept their lack of control over problematic situations. An alternative explanation could reflect the conceptual overlap among some ER strategies, which suggests the presence of associations among theoretically distinct scales. This point is especially reinforced by the evidence that, among young adults, other-blame showed significant bivariate associations with all the symptoms analyzed, although this effect disappeared when partializing for each ER strategy.

The evidence that some adaptive emotion regulation strategies (e.g., positive refocusing and low refocus on planning) were relevant in explaining low adult psychopathology, different from what was found in adolescents, may be explained by research on the refinement of the emotion regulation repertoire across the lifespan [34,103]. Some scholars claimed that the impacts of ER strategies on psychological functioning might be attributed to the increasing competence in individual and social emotion regulation observed in emerging adulthood, which typically results in the successful completion of developmental tasks (e.g., establishing stable relationships) [104] and higher emotional stability as compared to adolescents [101,105]. It is plausible that the differential relevance of adaptive emotion regulation strategies in predicting mental distress in adults and adolescents observed in the present work might reflect the aforementioned evidence that emotion regulation with growing age is more effective as a protective factor against psychological difficulties [106]. An additional explanation could reflect the context-dependent nature of adaptive ER strategies [6], as mentioned earlier in this study. Further, longitudinal studies are needed to further confirm this argument by investigating whether the unique contribution of cognitive emotion regulation strategies on mental distress changes depending on contexts and social or environmental resources. For example, some regulation strategies may have different meanings and thus different effects on mental health depending on local cultural expectations [107]. In this regard, some authors found no differences in the association between the use of suppression and internalizing symptoms in Western and Eastern samples [108], while other evidence suggested greater negative emotion for European participants employing this strategy and less negative emotion for participants with bicultural values [109]. Future work should use a longitudinal design to further examine whether the use of specific ER strategies may differently impact individual functioning depending on social and cultural contexts. This is especially true when considering the impacts of a challenging global event such as the COVID-19 pandemic, which, despite not being a central theme of the present study, critically affected individual mental health functioning [110]. Cumulative studies indicated a high prevalence of anxiety, depression, and sleep disorders among young adults [110,111] and adolescents [112] during the pandemic. In particular, adolescents’ difficulty in emotion regulation has been highlighted as one of the most impactful pandemic factors on mental distress [108].

Moreover, the present study contributed to our understanding of the role of gender in explaining psychological difficulties. Findings from the two studies revealed that, above the influence of ER strategies on these outcomes, women tended to report more psychopathological symptoms than men, with an increasing trend also observed for girls’ insomnia symptoms. This evidence provides additional support for gender differences with respect to internalizing symptoms [113] and insomnia in adolescents [114], as well as mental health in young adults [115]. Female participants also presented higher scores in some of the CERQ dimensions (e.g., self-blame), apparently supporting previous evidence on the greater likelihood of women to report engaging in emotion regulation strategies, as compared to men [40]. Future studies on the impacts of ER on psychological difficulties and insomnia should focus on the role of gender, especially considering the lack of vital information on how men regulate their emotions in the literature [40].

The present work clearly has some limitations. First, because of the cross-sectional nature of the two studies, causal inferences on the direction of the relationship between the use of emotion regulation strategies and psychopathological symptoms are precluded. Furthermore, the exclusive use of self-report assessments may have caused social desirability bias. Further studies would employ objective indicators when feasible (e.g., objective sleep duration). Another limitation of our study relates to the inability to make direct comparisons between the associations of emotion regulation (ER) and psychopathology found in adults and adolescents due to differences in the battery of self-report instruments employed to assess mental distress. The discrepancy in sample sizes between the two groups may also have influenced the ability to detect statistically significant associations in the adolescent group due to the lower statistical power. Future investigations addressing this gap are needed, e.g., by administering the same set of questionnaires in the two cohorts and conducting multigroup structural equation modelling (SEM) to statistically compare the magnitudes of the associations. Moreover, non-clinical and convenience samples were enrolled. As such, caution should be exercised when interpreting and generalizing the findings of our study, and future investigations are warranted to examine these relationships in clinical samples. Lastly, further research should examine other potential confounders not assessed in the present study (e.g., level of education, general health status, and electronic device use), which could affect the associations between specific ER strategies and psychopathological outcomes.

## 9. Conclusions

Despite the aforementioned limitations, the present study sheds new light on the limited body of evidence on the use of specific cognitive ER strategies in adolescence and young adulthood, as well as their associations with psychological difficulties observed during these life periods. The present research suggests that cognitive ER strategies are meaningful dimensions in the field of adolescent and adult psychopathology and allows us to draw some implications for clinical practice. First, the results suggest that the use of maladaptive strategies seems to be only dysfunctional, in terms of depression, anxiety, and insomnia symptoms in the case of adults, and in terms of internalizing and insomnia difficulties in the sample of adolescents. On the other hand, adaptive strategies are associated with reduced symptoms of depression, anxiety, and insomnia only in the sample of adults. Therefore, a reasonable direction could be to increase adaptive strategies and/or reduce maladaptive strategies when treating patients experiencing clinically relevant mental distress and sleep problems. Available intervention programs focused on the reduction in depression and anxiety [116], as well as internalizing symptoms [117], may benefit from incorporating emotion regulation training [118]. The same may be conducted for sleep intervention programs. Thus far, ER strategies have not yet been included or combined with the actual gold-standard treatment for insomnia, namely cognitive-behavioral therapy for insomnia (CBT-I), with only a few exceptions [119]. Recently, Cerolini and Lombardo (in press) [119] proposed a new eight-session therapeutic protocol combining four sessions targeting sleep difficulties through a standard CBT-I training [120] with four sessions focusing on emotion regulation ability and strategies [121]. For example, this may increase the ability to efficiently regulate emotions during the day, thus reducing the potential negative impact of a bad night of sleep and preventing the detrimental influences of emotion dysregulation on sleep activity. This may limit the perpetuation of a “vicious cycle”, in which poor sleep impairs ER, which in turn affects sleep, leading to further deterioration of emotional well-being [122].

In addition, other authors [123] observed that changes in ER over the course of many behavioral interventions predict changes in numerous clinically relevant psychopathological outcomes (e.g., diminished self-harm frequency) in adults. Moreover, the literature documents the effectiveness of psychological interventions to improve ER in youth and indicates that these improvements correlate with reduced psychopathology (e.g., depression) [124]. In conclusion, including interventions that directly target general regulatory skills can improve the effectiveness of psychotherapeutic programs by emphasizing the importance of tolerating negative emotions and increasing emotional self-efficacy [125], although conclusive evidence on the crucial role of ER protocols in predicting clinical improvement is still debated [126].

## Figures and Tables

**Table 1:** Descriptive statistics of the variables under study and independent sample t-tests across gender. Notes: Welch’s *t*-test was applied in the case of inequality of variances. Young adult group. * *p* < 0.05; ** *p* < 0.01; *** *p* < 0.001. Abbreviations: df, degrees of freedom; SD, standard deviation; BDI-II, Beck Depression Inventory-II scores; STAI-T, State–Trait Anxiety Inventory Trait Version scores; ISI, Insomnia Severity Index scores.

	Whole Sample	Men	Women		
	Mean (SD)	Mean (SD)	Mean (SD)	t (df)	Cohen’s d
Rumination	13.93 (3.53)	13.57 (3.18)	14.08 (3.67)	−1.34 (422)	−0.14
Catastrophizing	7.95 (2.92)	7.65 (2.91)	8.04 (2.91)	−1.27 (422)	−0.13
Self-blame	10.77 (2.95)	10.19 (2.67)	11.01 (3.04)	−2.59 (422) *	−0.27
Other-blame	8.47 (3.05)	8.76 (3.04)	8.34 (3.03)	1.29 (422)	0.13
Putting into perspective	12.57 (3.97)	11.90 (4.14)	12.77 (3.83)	−2.07 (422) *	−0.22
Acceptance	12.29 (3.09)	12.69 (3.00)	12.12 (3.13)	1.73 (422)	0.18
Positive refocusing	9.33 (3.61)	8.88 (3.65)	9.54 (3.57)	−1.71 (422)	−0.18
Positive reappraisal	14.44 (3.57)	14.82 (3.43)	14.31 (3.60)	1.34 (422)	0.14
Refocus on planning	15.38 (3.18)	15.57 (3.07)	15.31 (3.23)	0.77 (422)	0.08
STAI-T	44.95 (10.16)	42.28 (8.73)	46.03 (10.50)	−3.74 (278.14) ***	−0.38
BDI-II	9.75 (7.86)	7.96 (6.37)	10.46 (8.27)	−3.36 (305.13) **	−0.33
ISI	6.81 (4.71)	6.21 (4.63)	7.02 (4.73)	−1.59 (405)	−0.17

**Table 2:** Pearson’s correlation coefficients between predictor and criterion variables. Notes: * *p* < 0.05; ** *p* < 0.01; *** *p* < 0.001. Young adult group. Abbreviations: BDI-II, Beck Depression Inventory-II scores; STAI-T, State–Trait Anxiety Inventory Trait Version scores; ISI, Insomnia Severity Index scores.

	STAI-T	BDI-II	ISI
Rumination	0.319 ***	0.310 ***	0.238 ***
Catastrophizing	0.484 ***	0.387 ***	0.238 ***
Self-blame	0.494 ***	0.429 ***	0.293 ***
Other-blame	0.163 **	0.138 **	0.118 *
Putting into perspective	−0.203 ***	−0.176 ***	−0.165 **
Acceptance	−0.020	−0.033	−0.042
Positive refocusing	−0.345 ***	−0.255 ***	−0.178 ***
Positive reappraisal	−0.416 ***	−0.280 ***	−0.109 *
Refocus on planning	−0.407 ***	−0.284 ***	−0.081

**Table 3:** Final steps of hierarchical multiple regression analyses. Notes: Gender was included as a covariate. As gender was coded as a dummy variable (i.e., 0 = men; 1 = women), the corresponding regression coefficients are reported in the unstandardized form. All other coefficients are standardized partial regression coefficients. * *p* < 0.05; ** *p* < 0.01; *** *p* < 0.001. Young adult group. Abbreviations: BDI-II, Beck Depression Inventory-II scores; STAI-T, State–Trait Anxiety Inventory Trait Version scores; ISI, Insomnia Severity Index scores; SE, standard error.

Predictor Variable	BDI-II Score		STAI-T Score		ISI Score	
	β	SE	β	SE	β	SE
Rumination	0.152 **	0.046	0.150 ***	0.040	0.101	0.053
Catastrophizing	0.195 ***	0.046	0.252 ***	0.040	0.130 *	0.053
Self-blame	0.242 ***	0.046	0.267 ***	0.039	0.181 **	0.053
Other-blame	0.030	0.043	0.026	0.037	0.067	0.050
Putting into perspective	−0.017	0.048	0.044	0.041	−0.109 *	0.055
Acceptance	0.016	0.041	−0.034	0.036	−0.054	0.048
Positive refocusing	−0.118 *	0.046	−0.175 ***	0.039	−0.099	0.053
Positive reappraisal	−0.059	0.054	−0.145 **	0.046	0.042	0.062
Refocus on planning	−0.150 **	0.050	−0.212 ***	0.043	0.013	0.057
Gender	B = 1.697 *	0.713	B = 2.267 **	0.796	B = 0.654	0.494
Model summary						
*F*	(10, 413) 21.033 ***		(10, 400) 43.661 ***		(10, 396) 7.179 ***	
R-Squared	0.337		0.522		0.153	
Adjusted R-Squared	0.321		0.510		0.132	
Durbin–Watson	1.732		1.811		1.854	

**Table 4:** Descriptive statistics of the variables under study and independent sample t-tests across gender. Notes: Welch’s *t*-test was applied in the case of inequality of variances. Adolescent group. * *p* < 0.05; ** *p* < 0.01; *** *p* < 0.001. Abbreviations: df, degrees of freedom; SD, standard deviation; YSR, Youth Self Report internalizing scores; ISI, Insomnia Severity Index scores.

	Whole Sample	Boys	Girls		
	Mean (SD)	Mean (SD)	Mean (SD)	t (df)	Cohen’s d
Rumination	12.05 (4.12)	11.24 (4.07)	13.05 (3.97)	−3.51 (245) **	−0.44
Catastrophizing	9.33 (3.48)	9.12 (3.49)	9.58 (3.47)	−1.03 (242)	−0.13
Self-blame	10.17 (3.37)	9.78 (3.47)	10.65 (3.20)	−1.98 (235) *	−0.25
Other-blame	8.53 (3.15)	9.03 (3.19)	7.90 (2.99)	2.78 (235) **	0.36
Putting into perspective	12.84 (4.11)	12.64 (3.96)	13.07 (4.28)	−0.79 (233)	−0.10
Acceptance	11.43 (3.85)	11.60 (3.77)	11.22 (3.94)	0.78 (243)	0.10
Positive refocusing	11.32 (4.51)	11.66 (4.67)	10.90 (4.28)	1.29 (241)	0.16
Positive reappraisal	13.58 (3.90)	13.55 (3.69)	13.62 (4.16)	−0.13 (238)	−0.01
Refocus on planning	14.08 (4.19)	14.13 (4.18)	14.02 (4.22)	0.18 (237)	0.02
YSR	17.56 (11.17)	14.38 (9.57)	21.45 (11.77)	−5.25 (224.25) ***	−0.66
ISI	6.51 (4.81)	5.64 (4.80)	7.57 (4.63)	−3.24 (251) **	−0.40

**Table 5:** Pearson’s correlation coefficients between predictor and criterion variables. Notes: * *p* < 0.05; ** *p* < 0.01; *** p < 0.001. Adolescent group. Abbreviations: YSR, Youth Self Report internalizing scores; ISI, Insomnia Severity Index scores.

	YSR	ISI
Rumination	0.466 ***	0.379 ***
Catastrophizing	0.422 ***	0.386 ***
Self-blame	0.483 **	0.318 ***
Other-blame	0.044	0.081
Putting into perspective	−0.211 **	−0.125
Acceptance	0.102	−0.073
Positive refocusing	−0.259 ***	−0.156 *
Positive reappraisal	−0.254 ***	−0.220 **
Refocus on planning	−0.163 *	−0.186 **

**Table 6:** Final steps of hierarchical multiple regression analyses. Notes: Gender was included as a covariate. As gender was coded as a dummy variable (i.e., 0 = boys; 1 = girls), the corresponding regression coefficients are reported in the unstandardized form. All other coefficients are standardized partial regression coefficients. * *p* < 0.05; ** *p* < 0.01; *** *p* < 0.001. Adolescent group. Abbreviations: YSR, Youth Self Report internalizing scores; ISI, Insomnia Severity Index scores; SE, standard error.

Predictor Variable	YSR Score		ISI Score	
	β	SE	β	SE
Rumination	0.214 **	0.061	0.236 **	0.070
Catastrophizing	0.168 **	0.060	0.195 **	0.069
Self-blame	0.259 ***	0.065	0.113	0.075
Other-blame	0.064	0.055	0.063	0.063
Putting into perspective	−0.079	0.067	0.051	0.077
Acceptance	0.079	0.053	0.055	0.061
Positive refocusing	−0.109	0.060	−0.027	0.069
Positive reappraisal	−0.107	0.070	−0.121	0.080
Refocus on planning	−0.060	0.074	−0.156	0.085
Gender	B = 5.260 ***	1.201	B = 1.253 *	0.596
Model summary				
F	(10,211) 18.187 ***		(10,211) 8.589 ***	
R-Squared	0.463		0.289	
Adjusted R-Squared	0.437		0.256	
Durbin–Watson	1.824		1.959	

## Data Availability

The data presented in this study are available upon reasonable request by emailing andrea.zagaria@uniroma1.it.
